# Behavioral Activation Mobile App to Motivate Smokers to Quit: Feasibility and Pilot Randomized Controlled Trial

**DOI:** 10.2196/54912

**Published:** 2024-04-04

**Authors:** Belinda Borrelli, Y Kiera Bartlett, Daniel Fulford, Greg Frasco, Christopher J Armitage, Alison Wearden

**Affiliations:** 1 Center for Behavioral Science Research Henry M. Goldman School of Dental Medicine Boston University Boston, MA United States; 2 Manchester Centre for Health Psychology School of Health Sciences University of Manchester Manchester United Kingdom; 3 Department of Psychological & Brain Sciences Boston University Boston, MA United States; 4 Sargent College of Health & Rehabilitation Sciences Boston University Boston, MA United States; 5 Rafik B. Hariri Institute for Computing and Computational Science & Engineering Boston University Boston, MA United States; 6 Greater Manchester Patient Safety Research Collaboration National Institute for Health and Care Research Manchester United Kingdom

**Keywords:** smoking cessation, mobile app, motivation, depressed mood, depression, behavioral activation, negative affect, positive affect, quit smoking, health behavior change

## Abstract

**Background:**

Behavioral activation (BA) is an evidence-based treatment for depression that fosters engagement in values-based activities to increase access to positive reinforcement. Depressed mood has been shown to hinder smoking cessation.

**Objective:**

This study determined the feasibility and preliminary efficacy of a mobile app to motivate smokers to quit by using BA and integrating motivational messages to quit smoking.

**Methods:**

Adult smokers (N=56; mean age 34.5, SD 9.52 years) who were not ready to quit smoking within 30 days were recruited from advertisements and randomized to either 8 weeks of the BA app (set 2 values-based activities per week+motivational messages+feedback on changes in smoking, mood, and values-based activities) or the control group (no app; received resources for quitting smoking). All participants completed the baseline and end-of-treatment web-based questionnaires. Controls also completed weekly web-based assessments, and BA app participants completed assessments through the app.

**Results:**

There were no dropouts and only 2 participants in each condition did not complete the end-of-treatment questionnaire. The results demonstrated that it is feasible to recruit smokers who are unmotivated to quit into a smoking cessation induction trial: 86% (57/66) of eligible participants were randomized (BA app: n=27; control: n=29). Participants reported high levels of satisfaction: 80% (20/25) of participants said they would recommend the BA app, there were moderate-to-high scores on the Mobile App Rating Scale, and 88% (22/25) of participants rated the app 3 stars or higher (out of 5). There were high levels of BA app engagement: 96% (26/27) of participants planned activities, and 67% (18/27) of participants planned 7 or more activities. High engagement was found even among those who were at the highest risk for continued smoking (low motivation to quit, low confidence to quit, and high negative affect). The results provided support for the hypothesized relationships between BA constructs: greater pleasant activity completion was associated with greater positive affect (*b*=0.37, SE 0.21; 95% CI –0.05 to 0.79; *P*=.08), and greater positive affect tended to predict fewer cigarettes smoked the next day (*b*=–0.19, SE 0.10; 95% CI –0.39 to 0.01; *P*=.06). Additionally, a greater number of activities planned was associated with lower negative affect (*b*=–0.26, SE 0.15; 95% CI –0.55 to 0.04; *P*=.09). Overall, 16% (4/25) of BA app participants set a quit date versus 4% (1/27) among controls, and there were promising (but not significant) trends for motivation and confidence to quit.

**Conclusions:**

The findings suggest that a mobile app intervention can be made appealing to smokers who are unmotivated to quit by focusing on aspects most important to them, such as mood management. This theory-based intervention has shown some initial support for the underlying theoretical constructs, and further efficacy testing is warranted in a fully powered trial.

## Introduction

Currently available evidenced-based treatments are specifically designed for smokers who are ready to quit within 30 days, which constitute only 12% of smokers [1]. Evidence-based strategies are needed to reach the remaining population of unmotivated smokers and accelerate their motivation to quit before smoking-related illness occurs (or worsens).

Because unmotivated smokers are not likely to seek treatment, the majority of research has focused on integrating smoking cessation into infrastructures that are frequented by smokers, such as health care or worksites [2-6]. Mobile apps are another channel to reach smokers who are unmotivated to quit. However, while there are studies examining the effectiveness of mobile apps for smoking cessation [7], to our knowledge, there are no studies that focus on using mobile apps for motivating smokers who are not ready to quit. This is likely because it is difficult to encourage unmotivated smokers to use a smoking cessation app because the content is not personally relevant to them. Given prior research showing that the use of mobile apps increases with greater personal relevancy and perceived utility [8], it is important to find out what is important to unmotivated smokers, provide information on that topic using a mobile app, and then gradually weave in a smoking cessation intervention to increase intervention engagement and motivate quitting. This is consistent with the “foot-in-the-door” technique from social psychology [9]. For example, in 1 smoking cessation induction trial, smokers were offered asthma education on how to prevent asthma attacks in their children with asthma who were recently admitted to urgent care for an asthma exacerbation. The intervention largely focused on what they cared about most (preventing future asthma attacks in their children), but smoking cessation and how to quit were gradually woven into the intervention over time [10-13].

We previously conducted a series of focus groups with smokers who were unmotivated to quit and found that the most important thing on their minds was managing mood and stress, and that they would be therefore interested in a mobile app that could help them with mood management [14]. This is consistent with results from our large prior quantitative study with smokers unmotivated to quit, which showed a high prevalence of depression and depressed mood [15]. Therefore, focusing on things that are most important to unmotivated smokers (such as stress and mood) may increase app engagement and prime them to consider quitting smoking. To date, smoking cessation interventions using smartphone apps alone have not been successful [7]. One reason for this could be the lack of focus on content that would most interest unmotivated smokers.

Therefore, we developed a mobile app based on behavioral activation (BA) [16-18], hypothesizing that increases in positive affect and decreases in negative affect could prime unmotivated smokers to consider quitting. BA is an evidenced-based treatment for depression and depressed mood that has been successfully used in both psychiatric and medical populations [19-22] and has been shown to be superior to medication [19]. BA has also been shown to be effective for depression when delivered via a web-based platform [23]. BA purports that depression results from chronically low levels of, or loss of, positive environmental reinforcement. The lack of both pleasant events and positive reinforcement is associated with depression and negative avoidant coping behaviors (eg, smoking) [17]. BA improves mood through strategies to increase activation (eg, setting goals to engage in activities that are consistent with personal values and goals), which in turn increases access to natural sources of positive reinforcement (eg, other people, hobbies, and activities) [24].

In a series of prior studies, we iteratively and collaboratively developed a BA-based mobile app with smokers who were unmotivated to quit, through focus groups, a “think aloud study,” and a 1-month single-arm usability trial [14]. This current study aimed to conduct a pilot randomized trial of the BA app with smokers who were unmotivated to quit in order to assess (1) the feasibility (eg, enrollment) of the BA app, (2) satisfaction and engagement with the BA app, (3) if unmotivated smokers complete BA app intervention activities despite risk factors for continued smoking (low motivation to quit, low confidence to quit and high negative affect), and (4) preliminary effectiveness of the BA app on smoking behavior and on motivation and confidence to quit smoking. We hypothesized that (1) smokers who are unmotivated to quit will engage with the BA app, (2) engagement with the BA app will be associated with lower negative affect and greater positive affect, and (3) those who are randomized to the BA app will show positive changes in smoking (eg, set quit date and reduce the number of cigarettes) and in smoking-related variables (greater motivation and confidence to quit).

## Methods

### Recruitment

The study was conducted in the United Kingdom. Potential participants were recruited through advertisements using a company specializing in the recruitment of research participants (Propeller Research). Members in their database were sent a brief web-based screening survey, and those who passed the initial screening were then screened via telephone by our staff. Participants were told that they did not have to want to quit smoking to be in the study and that the study focused on improving the well-being of smokers. They were also told that the study would provide them with tips and resources to quit smoking in case they became motivated to quit smoking. Eligible participants who completed a web-based consent form were asked to complete a web-based baseline questionnaire. The inclusion criteria were (1) aged 18 years or older, (2) current smoker (>100 in lifetime and ≥3 cigarettes per day), (3) not planning to quit smoking within 30 days, and (4) owned and regularly used an Android or iPhone and had previously downloaded and used an app. Exclusion criteria were (1) inability to read, speak, or understand English to a sufficient level to understand the information sheet and app; (2) self-report that they do not wish to stop smoking at any point in the future; and (3) current involvement in any other research about smoking.

### Ethical Considerations

The study was approved by the University of Manchester Research Ethics Committee 5 (reference 2017-0128-2605). Participants were sent both the participant information sheet and the consent statements to consider during the recruitment process (Multimedia Appendix 1). All participants then completed a web-based consent form prior to completing the baseline questionnaire. Data were collected and stored in accordance with the Data Protection Act and the university-approved Data Management Plan. All data are anonymous, identified only by a participant number. Participants received a £20 (US $25.30) love2shop voucher for completing the follow-up questionnaire (valid at a range of high street stores and retailers; Multimedia Appendix 2).

### Procedure

#### Design

**Figure 1:**
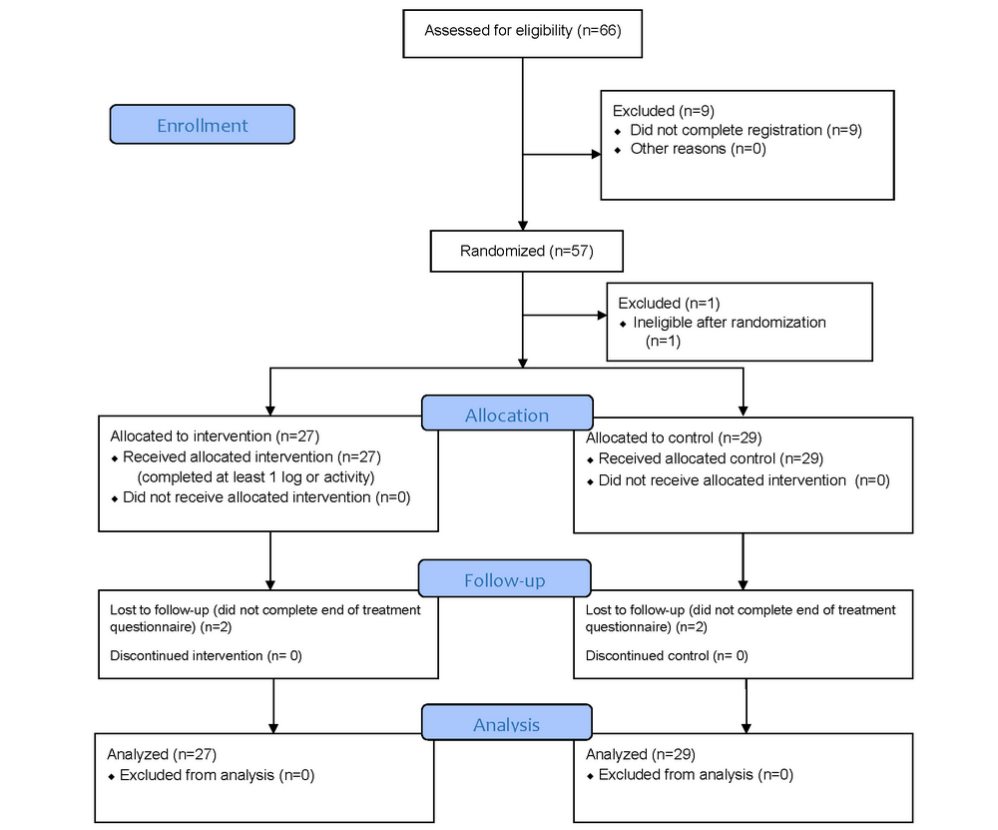
CONSORT (Consolidated Standards of Reporting Trials) diagram.

Participants were randomized to either the BA app or to the control condition using a parallel 1:1 allocation ratio. The random sequence of the BA app or control (represented by a 1 or a 2) was generated by GraphPad [25] in blocks of 10 participants. As participants consented, they were allocated to the next available number in the sequence. Study staff could not be masked to treatment conditions because some participants needed assistance with downloading the app. These staff were not involved in collecting the outcome data. Participants were allocated to either use the BA app for 8 weeks or to receive an information sheet with resources related to quitting smoking (control). Both groups completed weekly assessments and received the same information and resources about quitting (either through the app for the BA app group or as an email attachment for the control group). Participant flow is outlined in Figure 1.

#### BA App Intervention

Programming for the mobile app was conducted by the Software and Innovation Lab at Boston University. If needed, research assistants guided participants with app downloads from the Google Play Store or Apple Store (via phone consultation). Once downloaded, participants were led through the app’s tutorial on its features and on the intervention to ensure that staff were not involved in the intervention. The BA app helped participants identify, schedule, and complete values-based activities. Specifically, participants identified their values (eg, doing things with others, volunteer work, hobbies, being spiritual, and community activism) through the app, which allowed the app to suggest activities consistent with these values (algorithms available upon request). The app then encouraged participants to set at least 1 activity goal in each of 2 categories once per week: “pleasurable activities” and “challenging and meaningful activities.” Pleasurable activities were defined as those that provide a short, pleasant break from the pace of life, such as reading, taking baths, or watching movies [26]. Challenging activities were defined as those that absorb the mind, require concentration, and promote a sense of accomplishment (eg, engaging in a hobby or skill) and a feeling that “time flies” when engaged with that activity [26]. Meaningful activities were defined as those in which people feel like they are making a difference or activities that involve spiritual endeavors (eg, volunteer work and attending religious services) [26]. Each week, participants scheduled their chosen activities using the calendar feature in the app. The app also allowed participants to set “custom” activities (outside of the ones suggested by the app) and subgoals if the activity was too large of a goal for the week (eg, “learn a new language”). Participants received app notification “reminders” on the days they had scheduled the activities and were also prompted to record whether or not they completed the activity on the scheduled day. Participants received “trophies” for progress on activities, which were displayed on a rewards screen on which the number of cigarettes they smoked that week and mood level was also displayed.

The identification, scheduling, and completion of values-based activities were used as a “foot-in-the-door” approach to provide motivational messages about smoking, previously developed from focus groups with smokers who were unmotivated to quit and designed to address barriers to quitting and myths about smoking cessation medications [14]. The messages were delivered through “app notifications,” and the frequency of delivery was calibrated to participants’ level of motivation to quit, which was assessed weekly through the app. If participants were planning on quitting within 30 days, they were sent notifications to review the “Resources to Quit” sections within the app which listed evidenced-based smoking cessation apps, quitlines, and local resources for quitting. Participants who were motivated to quit within 30 days were additionally sent smoking cessation messages from a different message bank within the app that focused on helping them plan a quit day and strategies for quitting.

#### Control

Control participants received an information sheet with resources related to quitting smoking, for example, links to smoking cessation apps and information about stop-smoking medications. This was the same information that was available to the BA app group, on the resources page with the app.

### Measures

#### Overview

All participants were asked to complete a web-based baseline and end-of-treatment questionnaires for which they received one £20 (US $25.30) gift card. Both groups completed brief weekly surveys: control participants were emailed a link to a web-based survey and BA app participants answered the daily and weekly questions through the app.

#### Participant Characteristics

Gender, age, level of education, ethnicity, household income, and employment status were assessed. Nicotine dependence was assessed with a single item from the Fagerström Test for Nicotine Dependence [27], which is highly correlated with the full scale [28].

#### Satisfaction With the BA App

The Mobile App Rating Scale (MARS), which has shown excellent internal consistency and reliability [29], was used to assess app quality, perceived impact of the app, and app satisfaction. The MARS app quality scale consisted of 4 subscales (engagement, functionality, aesthetics, and information), each rated on a 1-5 scale (higher scores=increasing satisfaction). The MARS Perceived Impact subscale was measured on a 1-5 scale (1=strongly disagree to 5=strongly agree) and was adapted to the content of the BA app (eg, the app increased my knowledge of quitting smoking and the app increased my motivation to quit smoking). Participants were asked to give their “star rating” as a measure of their satisfaction on a 1-5 scale (1=one of the worst apps I have used through 5=one of the best apps I have used), as well as report if they would recommend the app to people who might benefit from it (1=not at all to 5=definitely).

#### Engagement With the App

Participants set weekly values-based activity goals through the app (“planned activities”) and reported on whether or not those activities were completed, also through the app on a weekly basis. The type of activity was also assessed (pleasant or challenging and meaningful) by the app. The app prompted participants to enter the above data and sent several notification reminders to do so.

#### Predictors of Engagement

Depressed mood was measured with the 10-item Center for Epidemiologic Studies Depression Scale (CES-D-10) [30,31]. Scores of 10 or higher indicate the presence of significant depressive symptoms [32]. Mood was also measured with the Positive and Negative Affect Schedule [33], which has 20 items that describe feelings and emotions (10 positive and 10 negative), each measured on a 5-point scale (not at all to extremely). The above variables were given at baseline, weekly, and at the end of treatment for both groups. In the BA app, the following variables were assessed on a daily basis: the number of cigarettes per day, motivation to quit smoking (1-10), confidence to quit smoking (1-10), and level of positive mood (happy, excited; 1-10 scale; higher=more positive) and negative mood (eg, sad, bored, or angry; 1-10 scale; higher=more negative).

#### Smoking-Related Variables

The following variables were assessed at baseline, weekly, and at the end of treatment: the number of cigarettes smoked per day, 7-day point prevalence (have you smoked any cigarettes at all in the past 7 days, even a puff?), motivation to quit (1-10 scale; 1= not at all motivated to quit, through 10=very much want to quit), and confidence to quit (1-10 scale; 1 is “not at all” confident to quit through 10 “very confident to quit”).

### Data Analysis

Descriptive analyses were conducted, and distributions of variables were inspected for violations of normality. Group differences were evaluated using chi-square and 2-tailed *t* tests (in all instances), for categorical and continuous variables, respectively. For the multilevel models, lead variables were created for each relevant outcome (eg, number of cigarettes) to assess prospective associations at the day and week level. Pleasant, meaningful and challenging, and the combined sum activities (ie, summed pleasant, meaningful, and challenging activities) were aggregated at the day and week level.

Due to the small sample of this pilot study, our primary analyses focused on describing trends (eg, examination of the direction of coefficients) rather than focusing on statistical associations. Analyses were conducted in R (version 4.2.3; R Foundation for Statistical Computing) using the *stats* package for 2-tailed *t* tests (in all instances), ANOVAs, and linear regressions and the *lme4* package [34] for multilevel model analyses. Group differences between the BA app and controls on baseline variables were examined with independent 2-tailed *t* tests (in all instances). Then, descriptive statistics were examined for the engagement and satisfaction of those in the BA app condition. While an intention-to-treat approach was not used given the preliminary nature of the study, differences in baseline variables of interest between participants who provided at least 33% complete weekly data for between-group analyses and 33% complete daily data for within-group analyses were examined.

Analyses first investigated satisfaction with the BA app through descriptive statistics and then investigated engagement with the BA app through both descriptive statistics and through prospective linear regressions focused on whether or not those at greatest risk for continued smoking at baseline (ie, negative affect, low confidence, and low motivation) would engage with the intervention (activity completion). Second, multilevel modeling, which accounts for the clustering of repeated observations, was used to examine the within-person effect or the degree to which the outcome of interest was predicted by a given person’s deviation from their typical level. Among those within the BA app group, concurrent (same day or week) and prospective (next day or week) within-person analyses of affect, motivation, and confidence to quit; activity planning and completion; and smoking were examined. Lastly, changes in key outcome variables (eg, cigarettes smoked, motivation, confidence, and affect) from pre- to postintervention were examined by group using general linear models.

## Results

### Preliminary Analyses

As shown in Figure 1, smokers who were not motivated to quit were willing to enroll in our trial: of those who were eligible, 86% (57/66) of participants consented and were randomized to either the BA app (n=27) or to the control group (n=29). Only 2 people in each condition did not complete the end-of-treatment questionnaire (Figure 1). Participant characteristics are displayed in Table 1. Participants smoked an average of 13.4 cigarettes per day, and 28 (50%) out of 56 participants reported smoking their first cigarette within 30 minutes of waking. Participants had low levels of both motivation to quit (mean 4.3, SD 2.2) and confidence to quit (mean 3.8, SD 2.4), as well as high levels of depressed mood (CES-D-10: mean 9.0, SD 5.4). There were no significant differences between groups on any baseline variable (all *P*>.05), and 7 (12%) out of 56 participants had >33% missing weekly data. There were no significant differences in demographics or smoking behavior between those with and without missing data. There were no reported side effects, technical problems, or privacy breaches during the course of the study.

**Table 1:** Participant demographics, smoking behavior, and mood.

Variable		Control (n=29)	BA^a^ app (n=27)	Overall (N=56)	Tests of difference, (N=56)			*P* value
					Chi-square (*df*=1)	*t* test (*df*=54)		
Age (years), mean (SD)		34.24 (8.99)	34.85 (10.23)	34.54 (9.52)	N/A^b^	0.24	.81	
Female, n (%)		21 (72)	20 (74)	41 (73)	0.02	N/A	.89	
**Race, n (%)**								
	Asian	2 (7)	3 (11)	5 (9)	0.01	N/A	.93	
	Black	2 (7)	1 (4)	3 (5)	0.01	N/A	.99	
	White	24 (83)	20 (74)	44 (79)	0.63	N/A	.43	
	Other	1 (3)	3 (11)	4 (7)	0.35	N/A	.55	
Less than university education, n (%)		11 (38)	11 (41)	22 (39)	0.05	N/A	.83	
Cigarettes smoked per day, mean (SD)		12.14 (6.93)	14.74 (10.39)	13.39 (8.79)	N/A	1.11	.27	
Confidence to quit, mean (SD)		3.93 (2.53)	3.78 (2.38)	3.86 (2.44)	N/A	0.23	.82	
Motivation to quit, mean (SD)		4.62 (2.29)	4 (2.17)	4.32 (2.23)	N/A	1.04	.30	
First cigarette within 30 minutes of waking, n (%)		15 (52)	13 (48)	28 (50)	0.72	N/A	.79	
CES-D^c^, mean (SD)		8.55 (4.72)	9.48 (6.14)	9 (5.42)	N/A	0.64	.53	
CES-D ≥10, n (%)		9 (31)	12 (44)	21 (38)	1.07	N/A	.30	
PANAS^d^ positive mood, mean (SD)		31.47 (8)	31.18 (7.21)	31.33 (7.54)	N/A	0.14	.89	
PANAS negative mood, mean (SD)		20.06 (7.02)	20.98 (8.68)	20.20 (7.80)	N/A	0.44	.66	

^a^BA: behavioral activation.

^b^N/A: not applicable.

^c^CES-D: Centers for Epidemiological Studies, Depression Scale.

^d^PANAS: Positive and Negative Affect Schedule.

### Mobile App Satisfaction and Engagement

#### Satisfaction

Satisfaction scores on the MARS subscales were within the “acceptable to very good” range (Table 2). Participants indicated moderate to strong agreement regarding the perceived impact of the app on awareness, knowledge, positive attitude toward quitting, intention to quit, further help seeking, and willingness to reduce their smoking (Table 2). The majority of the participants (21/25, 80%) said they would recommend the app to several people, many people, or “everyone” (Table 2). Three participants rated the app as 2 stars or less, while 10 participants gave it a 3-star rating, 11 gave it a 4-star rating, and 1 gave it a 5-star rating.

**Table 2:** Satisfaction with the BA^a^ app.

Variable			BA app group (n=25)
**App quality subscale, mean (SD)**			
	Engagement	3.18 (0.65)	
	Functionality	4.06 (0.67)	
	Aesthetics	3.68 (0.64)	
	Information	3.86 (0.41)	
	Overall	3.69 (0.49)	
**Perceived impact of the app, mean (SD)**			
	The app increased my awareness of the importance of setting values-based activities to help me think about quitting smoking	3.64 (1.11)	
	The app increased my knowledge of quitting smoking	3.44 (1.19)	
	The app has made my attitude toward quitting smoking more positive	3.36 (1.25)	
	The app has increased my motivation to quit	3.16 (1.41)	
	The app would encourage me to seek further help to quit smoking (if I decided to quit)	3.76 (1.09)	
	Use of this app will decrease my smoking	2.96 (1.37)	
**Recommend to people, n (%)**			
	Not at all	1 (4)	
	Very few people	4 (16)	
	Several people	12 (48)	
	Many people	5 (20)	
	Everyone	3 (12)	
**Overall star rating, n (%)**			
	1 Star	1 (4)	
	2 Stars	2 (8)	
	3 Stars	10 (40)	
	4 Stars	11 (44)	
	5 Stars	1 (4)	

^a^BA: behavioral activation.

#### Engagement With the App and Predictors of Engagement

Participants demonstrated high levels of engagement with the app, despite being not motivated to quit smoking. All participants in the BA app group planned at least 1 activity. A total of 253 activities were planned (132 pleasant and 121 meaningful and challenging); 26 (96%) out of 27 participants planned at least 1 activity after the first week and 19 (70%) out of 27 participants planned 7 or more activities over the course of the 8-week intervention. A total of 138 activities were completed (68 pleasant and 70 meaningful and challenging). Four participants did not complete any activity (either pleasant or challenging and meaningful).

Next, we explored whether or not those at risk for continued smoking (lower motivation and confidence to quit; high negative affect) would actually engage with the intervention (eg, complete values-based activities; Table 3). Lower motivation to quit at baseline was prospectively associated with more total activities completed during the intervention (*b*=–0.71, SE 0.26; 95% CI –1.23 to –0.20; *P*=.01), more pleasant activities completed during the intervention (*b*=–0.30, SE 0.14; 95% CI –0.57 to –0.02; *P*=.04), and more meaningful and challenging activities completed during the intervention (*b*=–0.42, SE 0.14; 95% CI –0.70 to –0.14; *P*=.01). Lower confidence to quit at baseline was prospectively associated with a greater number of total activities completed during the intervention (*b*=–0.53, SE 0.25; 95% CI –1.03 to –0.04; *P*=.03) and more pleasant activities completed during the intervention (*b*=–0.33, SE 0.12; 95% CI –0.57 to –0.08; *P*=.01), but not more meaningful and challenging activities completed. Lower negative affect at baseline was prospectively associated with a greater number of total activities completed during the intervention (*b*=–1.47, SE 0.69; 95% CI –2.82 to –0.12; *P*=.03), more meaningful and challenging activities completed during the intervention (*b*=–0.86, SE 0.38; 95% CI –1.60 to –0.12; *P*=.02), and a trend for more pleasant activities completed during the intervention (*b*=–0.61, SE 0.36; 95% CI –1.36 to 0.11; *P*=.10; not statistically significant).

**Table 3:** Prediction of intervention engagement from baseline variables (n=25).

Predictors		Total number of activities completed			
		*b*	SE	95% CI	*P* value
**Total number of activities completed**					
	Baseline positive affect	0.02	0.90	–1.75 to 1.79	.98
	Baseline negative affect	–1.47	0.69	–0.12 to –2.82	.03
	Baseline motivation	–0.71	0.26	–1.23 to –0.20	.01
	Baseline confidence	–0.53	0.25	–1.03 to –0.04	.03
**Number of pleasant activities completed**					
	Baseline positive affect	0.07	0.46	–0.83 to 0.98	.88
	Baseline negative affect	–0.61	0.36	–1.36 to 0.11	.10
	Baseline motivation	–0.30	0.14	–0.57 to –0.02	.04
	Baseline confidence	–0.33	0.12	–0.57 to –0.08	.01
**Meaningful and challenging activities completed**					
	Baseline positive affect	–0.05	0.50	–1.03 to 0.93	.92
	Baseline negative affect	–0.86	0.38	–1.60 to –0.12	.02
	Baseline motivation	–0.42	0.14	–0.70 to –0.14	.01
	Baseline confidence	–0.21	0.15	–0.49 to 0.08	.16

### Relationships Between Activities and Predictors (BA App Group Only)

Participants who completed a greater number of pleasant activities tended to report greater positive affect on the same day (*b*=0.37, SE 0.21; 95% CI –0.05 to 0.79; *P*=.08). Participants who reported higher levels of positive affect on 1 day tended to smoke fewer cigarettes the next day (*b*=–0.19, SE 0.10; 95% CI –0.39 to 0.01; *P*=.06). On days in which individuals experienced greater negative affect (ie, worsened negative affect), they smoked more cigarettes the next day (*b*=0.16, SE 0.08; 95% CI 0.01-0.32; *P*=.04). A greater number of activities planned in a given week, compared to an individual’s typical level, tended to predict lower negative affect (ie, more favorable negative affect) the same week (*b*=–0.26, SE 0.15; 95% CI –0.55 to 0.04; *P*=.09), although this was not significant. The total number of activities and activity types did not significantly predict the number of cigarettes smoked the next day.

### Smoking-Related Variables and Group Differences

Table 4 displays changes in smoking-related variables from baseline to follow-up for both groups. There was a trend for fewer cigarettes smoked per day across the intervention period (*b*=–0.19, SE 0.11; *P*=.07; not statistically significant) for both groups. There was no group main effect (*b*=1.67, SE 1.25; *P*=.19) or difference in slope between groups (*b*=0.02, SE 0.17; *P*=.91). Across both groups, motivation to quit increased significantly over time (*b*=0.19, SE 0.06; *P*=.01). There was also a main effect of group (*b*=1.61, SE 0.42; *P*=.01), indicating that participants in the BA app group had higher levels of motivation to quit across the intervention period. The slope for group was not significant, however, suggesting no group differences in the rate of increase in motivation over time (*b*=–0.05, SE 0.09; *P*=.63). While there was a trend for higher confidence to quit across the intervention period in the BA app group (*b*=0.90, SE 0.51; *P*=.08; not statistically significant), confidence did not change significantly during the intervention period between groups (*b*=0.07, SE 0.05; *P*=.19).

At the end of the intervention, 4 of 25 (16%) participants in the app group and 1 participant of 27 (3.7%) in the control group set a quit date (χ _1_^2^=2.25; *P*=.13). Two (25%) of 8 participants in the app group and 1 (8%) of 12 participants in the control group reported that they planned to quit in the next 30 days (χ_1_^2^=1.05; *P*=.31).

**Table 4:** Group differences between the BA^a^ app condition and the control condition.

Variable	BA app group, mean (SD)^b^			Control, mean (SD)^c^			Changes in baseline to follow-up				Group interaction		
	Baseline	Follow-up	Baseline		Follow-up	*F* (*df*=1)		*P* value	η^2^	*F* (*df*=1)		*P* value	η^2^
Motivation	3.92 (0.45)	5.76 (0.49)	4.67 (0.43)		5.56 (0.47)	13.13		.01	0.21	1.60		.21	0.03
Confidence	3.88 (0.50)	4.68 (0.5)	3.93 (0.48)		4.26 (0.48)	2.22		.14	0.04	0.38		.54	0.01
CES-D^d^	9.12 (5.90)	9.76 (5.19)	8.11 (4.59)		10.56 (7.01)	2.96		.09	0.06	1.01		.32	0.02
Positive affect (PANAS)^e^	3.19 (0.15)	2.97 (0.18)	3.15 (0.14)		3.10 (0.17)	1.10		.30	0.02	0.36		.55	0.01
Negative affect (PANAS)	2.04 (0.16)	1.95 (0.16)	1.98 (0.15)		2.11 (0.16)	0.02		.90	<0.01	0.77		.38	0.02

^a^BA: behavioral activation.

^b^BA app group (n=25).

^c^Control condition (n=27).

^d^CES-D-10: Centers for Epidemiological Studies, Depression Scale 10-item Version.

^e^PANAS: Positive and Negative Affect Schedule.

## Discussion

To our knowledge, there are no mobile apps that focus on motivating unmotivated smokers to quit, which comprise the majority of smokers. Our study filled this gap by developing and pilot-testing a theory-based mobile app that focuses on motivating smokers to quit by directly targeting mood-based barriers to quitting. Our main findings were (1) smokers who were unmotivated to quit were willing to enroll in our study (of those eligible to participate, 57/66, 86%, were randomized), (2) participants reported high levels of satisfaction and engagement with the intervention (ie, scheduling and completing activities), (3) those at highest risk for continued smoking (low motivation to quit, low confidence to quit, and high negative affect) demonstrated high levels of intervention engagement, and (4) the hypothesized associations between theory-based BA constructs (eg, activity scheduling and completion) and outcomes (positive and negative affect and smoking behavior) were in the expected direction. As expected, due to the small sample size, there were no significant differences between the groups on smoking behavior, but there were promising trends in the hypothesized direction regarding the number of cigarettes smoked, setting a quit date, and motivation and confidence to quit. The above findings suggest that mobile app interventions can be made appealing to smokers who are unmotivated to quit and warrant further efficacy testing.

Feasibility studies are used to determine whether an intervention is appropriate for further testing, particularly when there are few or no published studies on a particular intervention technique [35]. Our study met the criteria for intervention feasibility outlined by Bowen et al [35]. Specifically, we demonstrated acceptability (satisfaction, engagement, and no dropout), demand (enrollment rate and use of app), implementation (successful execution), practicality (ease and quality of implementation, and low burden on patients), integration (fit into daily life), and limited efficacy (promising effects of the program on key variables). Therefore, we believe that there is sufficient evidence to warrant further testing in a larger trial.

The novel aspect of the app is that it takes a “foot-in-the-door” approach by focusing on aspects that we found matter most to unmotivated smokers (stress and mood management) with the idea that addressing these risk factors for continued smoking will increase smokers’ receptivity to pushed content on smoking cessation. While it may seem counterintuitive to enroll unmotivated smokers into a smoking cessation app, we were able to demonstrate that our approach is feasible, given our high rate of enrollment and no dropout. One other study has also demonstrated high enrollment of unmotivated smokers by focusing on aspects that matter most to them, and then weaving in smoking cessation messages [12]; however, there was no digital component in this study. Additionally, our sample was comprised of smokers with high levels of depressed mood (21/56, 38% scored above the cutoff for depression on the CES-D) and low motivation and confidence to quit, indicating that we were able to attract smokers who are at risk for continued smoking.

Our results showed moderate to high levels of satisfaction and high levels of engagement with the intervention (planning and completing activities). High levels of engagement with the app also mean that these smokers, who were unmotivated to quit, received our intermittent, pushed messages about smoking cessation; messages that they would not otherwise receive. The messages focused on key barriers to quitting and were based on state-of-the-art motivational approaches [12,36] as well as our previous mixed methods research with the target population [14]. We believe that this approach was successful because there were no study dropouts.

Although counterintuitive, those with greater risk factors for continued smoking (low motivation to quit, low confidence to quit, and high negative affect) completed a greater number of a variety of activities over the course of the study (ie, engaged with the hypothesized active ingredients of the intervention). These findings have several implications. First, just because smokers are unmotivated to quit, or have high risk factors for continued smoking, does not mean that they are not willing to engage with an intervention. This finding lends support to continue with future research in developing digital behavior change solutions for those who are unmotivated to change. Second, these associations were prospective and provided support for the theoretical model underpinning the intervention. Finally, this finding dovetails with a previous study with smokers, where smokers who were unmotivated to quit were more likely to quit with an intensive motivational interviewing intervention than a less intensive one [13].

Consistent with BA theory, we hypothesized that completing a greater number of activities would lead to higher positive affect and lower negative affect and that positive changes in mood would be associated with less smoking. We found support for some but not all of these associations. We found that pleasant activities were associated with greater positive affect and that positive affect predicted smoking fewer cigarettes the next day. Although significance was borderline due to the small sample, the fact that these trends were based on a theoretical model specified a priori give support for future study of these relationships in a larger sample. Planning more activities was also associated with lower levels of negative affect (although only a trend), but lower levels of negative affect were significantly and prospectively associated with smoking fewer cigarettes. This set of findings indicates support for key relationships with the BA model and therefore warrants further investigation.

As with any pilot feasibility study, there are limitations in what can be concluded from the study. For example, the effectiveness of the app on smoking behavior cannot be determined due to lack of power, although there were promising trends in the hypothesized direction regarding intentions to quit. While we can conclude that the results are promising and warrant a larger trial, we cannot conclude that the cross-sectional and prospective relationships between hypothesized variables will be demonstrated in a larger trial. In general, our effects were small in size and should be interpreted with caution. The trial was also conducted in the United Kingdom with a relatively racially and ethnically homogeneous population, so it is unclear if smokers who are unmotivated to quit in other countries and smokers of other races and ethnicities would find the app equally satisfactory or if they would engage with the app to the same extent. Additionally, participants and study staff could not be masked to treatment conditions, given the study design and digital platform. Finally, although satisfaction scores were in the “moderate to high” range, future studies should do qualitative work postintervention to learn about how to increase satisfaction with the app.

These limitations should be viewed in the context of innovation. Innovation regarding intervention approaches for unmotivated smokers has stalled. The vast majority of digital interventions include content and features that are tailored for those who are motivated to quit. On a broader scale, digital interventions in general have not been sufficiently leveraged to target those who are not motivated to change. This study presents progress in this direction and could potentially serve as a paradigm for others to blend social psychology principles (eg, foot-in-the-door approach) with evidenced-based treatments (BA) to motivate change across different areas of health using digital platforms.

## Appendix

Multimedia Appendix 1 Study protocols.

Multimedia Appendix 2 CONSORT-EHEALTH checklist.

## Abbreviations

BA: behavioral activation
CES-D-10: 10-item Center for Epidemiologic Studies Depression Scale
MARS: Mobile App Rating Scale

## Data Availability

The data sets generated and analyzed during this study are available from the corresponding author on reasonable request.
