# Supplementary material for: Behavioral Activation Mobile App to Motivate Smokers to Quit: Feasibility and Pilot Randomized Controlled Trial
Source: JMIR Form Res. 2024 Apr 4;8:e54912. doi: 10.2196/54912 (PMC11027050; doi:10.2196/54912)
Supplement: Multimedia Appendix 1 [file formative_v8i1e54912_app1.docx]

**Study Protocol**

Standard Operating Procedure (SOP)

**Before this SOP** – the details of potential participants will have been sent and downloaded securely into a password protected spreadsheet ‘Contacts_Pilot trial.xlsx’. Each person has been allocated a screening number (this will become their participant ID). All researchers will have access to the shared drive on the UoM server and the shared folder in Google Drive.

New potential participants

Documents: Contacts_Pilot trial.xlsx, Monitoring_Pilot trial.xlsx, Contact_log.docx

1. For each new participant – put your initials in **“Researcher” in** Monitoring_Pilot trial.xlsx.

Call or email to arrange an orientation call

a. If able to contact participant arrange the call [complete “**Screening_ID**” and “**Orientation call booked - DATE**” in Monitoring_Pilot trial.xlsx], complete the information on the Contact_log.docx and proceed to step 2.

b. Call and leave a message for the participants (completing details in Contact_log.docx) [enter “NA” in **“Orientation call booked – DATE”** and **“Orientation call completed – DATE”** in Monitoring_Pilot trial.xlsx] and proceed to step 3.

2. Orientation Call [See Phone call scripts]

[Enter the date the call is completed into “**Orientation call completed – DATE”** Monitoring_Pilot trial.xlsx] and proceed to step 3.

3. Send out the link to the BL Q and consent form [Put the date into “**Link to BL and consent sent – DATE”** Monitoring_Pilot trial.xlsx]

Checking consent is received, and next actions

Documents: Monitoring_Pilot trial.xlsx, Select Survey, Allocation_sequence.xlsx, additional information for experimental participants, download instructions

1. Login to Select Survey Export ‘Smokers’ Health Study Pilot Trial Baseline Questionnaire’ as SPSS Condensed format. Save as SHSBL_today’s date (e.g. SHSBL_06_06_17). See ‘Exporting data from select survey.docx’ for screenshots.

a. If consent has been received - complete **“Consent and BL received – DATE”** in Monitoring_Pilot trial.xlsx, if reminder has not been needed enter “NA” **“BL Reminder sent – DATE”**  delete the yes or no in “**BL reminder need to send – AUTO”** , delete the number in “**BL time elapsed 2 – AUTO”** and proceed to step 2.

b. If consent has not been received - open Monitoring_Pilot trial.xlsx and send reminders (see Reminders.docx) where indicated with a ‘yes’ in **“BL reminder need to send – AUTO”**  and complete the date the reminder was sent in **“BL Reminder sent – DATE”** and delete the ‘yes’ in “**BL reminder need to send – AUTO”** in Monitoring_Pilot trial.xlsx and complete details on Contact_log.docx

c. If consent is still not received 3 days after the reminder has been sent **“BL time elapsed 2 – AUTO”** will show pink, mark the participant as a dropout and speak to the research team about requesting a substitute from Propeller Research.

2. When consent is received, project director open Allocation_Sequence.xslx and assign the participant the next consecutive randomiseID number by putting in their screening ID/ Ppt number and look at the allocation. Add the randomiseID to “**RandomiseID**” in Monitoring_Pilot trial.xlsx and enter either Exp or Cont into **“Group Allocation – “Exp” or “Cont”** in monitoring_Pilot trial.xlsx

3. Allocation call [See Phone call scripts]

a. If able to contact participant complete the allocation call [complete “**Allocation call completed - DATE**” lot trial.xlsx], complete the Contact_log.docx and proceed to step 4.

b. If the participant still cannot be reached after all attempts are completed on the Contact_log.docx then enter “NA” into **“Allocation call completed”** in Monitoring_Pilot trial.xlsx and proceed to step 4.

4.For participants in the experimental group send additional information sheet (Additional information for those in the experimental group_v1 09_03_17.docx) and appropriate download instructions (either Download instructions Android.docx or Download instructions iPhone.docx). For participants in the control group, attach the Information_Brochure.docx and send the link to the first online questionnaire. Complete **“Links sent (app or weekly questionnaire)- DATE”** in Monitoring_Pilot trial.xlsx

Day to day management of the trial

Documents: Monitoring_Pilot trial.xlsx, Select Survey , Contact_log.docx

1. Monitor participants progress through the trial

**If experimental**

i. Log into the study data site. Click ‘User’s Activity Counts’. Users who have the lowest activity will be displayed at the top. Look for anyone who has had no activity within the last 7 days.

ii. If every participant has activity in the last 7 days, no further action required.

iii. If there is a user (or users) who have no activity in the last 7 days, proceed to step 2.

iiii. if there is a user (or users) who have not set an activity or completed a weekly log for >14 days, we will need to manually send them notifications to complete their weekly log every Monday through the text message service. Open Text_Message_Notification.docx and send template via *txtmessenger* email service.

**If control**

a. Login to Select Survey. Export ‘Smokers’ Health Study Control Q [X]’ as SPSS Condensed format. Save as SHSCQ_[X] (e.g. SHSCQ_3) ([X] = relevant week). See ‘Exporting data from select survey.docx’ for screenshots.

b. Complete the appropriate date in the complete box (e.g. “**Q1 complete – DATE**”, “**Q2 complete – DATE**”…) in Monitoring_Pilot trial.xlsx

b. Check the Monitoring_Pilot trial.xlsx spreadsheet. Due dates for weekly questionnaires should be automatically filled, look for due boxes filled with ‘Yes’. When a questionnaire link is sent complete the appropriate date in the sent box (e.g. “**Q1 sent – DATE**”, “**Q2 sent – DATE**”…) and delete the ‘Yes’ or ‘no’ from the **Qx – due – AUTO.**

2. If no data is received, check Contact_log.docx for previous catch-up calls. Participants receive a catch-up call after 7 days of no activity – however if they remain inactive we probably don’t want to be calling them every 7 days. For continually inactive participants there are 4 potential contact dates for catch-up calls at 9, 24, 40 and 50 days following the link being sent – participants only switch to this schedule when they have been continually inactive for more than 7 days.

i) If no previous catch-up call has been made, check contact details for relevant participants in Contacts_Pilot trial.xlsx and try to have a catch-up call with the participant [See Phone call scripts] and complete details in Contact_log.docx (add new Catch up calls as needed in the log, number consecutively) and Monitoring_Pilot trial.xlsx spreadsheet.

ii) If a previous catch up call has been made less than 7 days ago – no further action required

iii) If a previous catch-up call has been made more than 7 days ago, this participant may need to be put onto the 2 weekly catch-up call schedule check **“**Catch up CALL 1/2/3/4/5 Complete - DATE**”** in Monitoring_Pilot trial.xlsx to determine when contact is required, following the 9, 24, 40, 50 day schedule.

If unable to contact:

1. For experimental: ensure all the details of attempts are recorded in Contact_log.docx and Monitoring_Pilot trial.xlsx spreadsheet and email out the App_usage_email_for_experiemtnal.docx.
2. For control: ensure all the details of attempts are recorded in Contact_log.docx and Monitoring_Pilot trial.xlsx spreadsheet. If participant has been inactive for 7 days email their newest weekly questionnaire link in the Catch_up_email_control_group.docx format and text message. If continually inactive (>7 days) email the weekly questionnaire as normal alongside text message.

2 months from the app being downloaded or 5 days from Q8 link being sent

Documents: Monitoring_Pilot trial.xlsx, Select Survey, Contacts.xlsx, Uninstall instructions

1. The **“Link to EoT Q due – AUTO”** will show when the EoT is due to be emailed. For experimental, the EoT is calculated automatically as 56 days from the **“App downloaded – DATE”**. For control, the EoT is calculated as 5 days from **“Q8 due – AUTO”**. If control complete their week 8 questionnaire prior to the **“Link to EoT Q due – AUTO”**, delete this date and type **“NA”** and move on to step 2.

2. When **“Link to EoT Q due – AUTO”** shows today’s date in Monitoring_Pilot trial.xlsx, send a link to the correct EoT Q Control: / Experimental:, put the date in “**Link to EoT Q sent – DATE”.** Also send text message notification to participant to let them know that their final link has been emailed.

2. Login to Select Survey). Export ‘Smokers’ Health Study EoT’ as SPSS Condensed format. Save as SHSEoT_[today’s date] (e.g. SHSEoT_10_08_17). See ‘Exporting data from select survey.docx’ for screenshots.

a. If EoT received - **Complete “EoT received by UoM – DATE”** in Monitoring_Pilot trial.xlsx. Check the address in Contacts_Pilot trial.xlsx and send the vouchers out to participants [complete **“Voucher numbers”** from the back of the love2shop voucher , **“Voucher sent (with uninstall details if necessary) – DATE”** and **“Tracker number”** from the Royal mail special delivery sticker in Monitoring_Pilot trial.xlsx]

ii) If EoT is not received – Check whether **“1^st^ Reminder for EoT Q due – AUTO”** shows ‘Yes’ in Monitoring_Pilot trial.xlsx – if it does, send the reminder (see Reminders.docx) and complete the date. Complete details in Contact_log.docx.

iii) If EoT is not received 3 days after the reminder is sent continue to try and contact up to 5 times over the next 2 weeks. Each attempt to contact should be added to Contact_log.docx and the total number of attempts noted in **“Number of attempts to contact – NUMBER”** in Monitoring_Pilot trial.xlsx, Once this reaches 5 it will show pink and no further attempts should be made.

Marking a participant as a dropout

Documents: Monitoring_Pilot trial.xlsx

As outlined above: If a participant fails to respond after BL or EoT reminders they should be marked as a dropout

1. Select the corresponding participant row in Monitoring_Pilot trial.xlsx, change the fill to light grey.

2. Complete details of the date, the point in the study that the participant dropped out and details of the contact attempts in the participant’s **Contact_log.docx**

3. If the participant drops out before they have returned their completed consent form – discuss with the research team when to request additional numbers from Propeller.


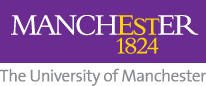


Smoker’s Health Study

Resources to Quit

You don’t have to be planning to quit to take part in the Smoker’s Health Study, but if you would like to learn more about quitting, and the resources that are available should you want to quit, see below

Ready to quit?

There are lots of options available to give you the support you need, you can find out more about the available options at: <http://www.nhs.uk/livewell/smoking/Pages/stopsmokingnewhome.aspx>

Or why not try one of the below:

- - Contact your GP or local stop smoking service for more information and support
  - Find a service near you. Visit <http://www.nhs.uk/service-search> and enter ‘Stop smoking services’ into the ‘Find’ box and your postcode into the ‘Location’ box
  - Call the Smokefree National Helpline free on 0300 123 1044 to speak to a trained, expert adviser. Lines are open weekdays 9am-8pm, and 11am to 4pm weekends
  - Try an evidence-based app. We’ve rounded up apps from around the world that can provide you with evidence-based support, search for them in your app store:

| 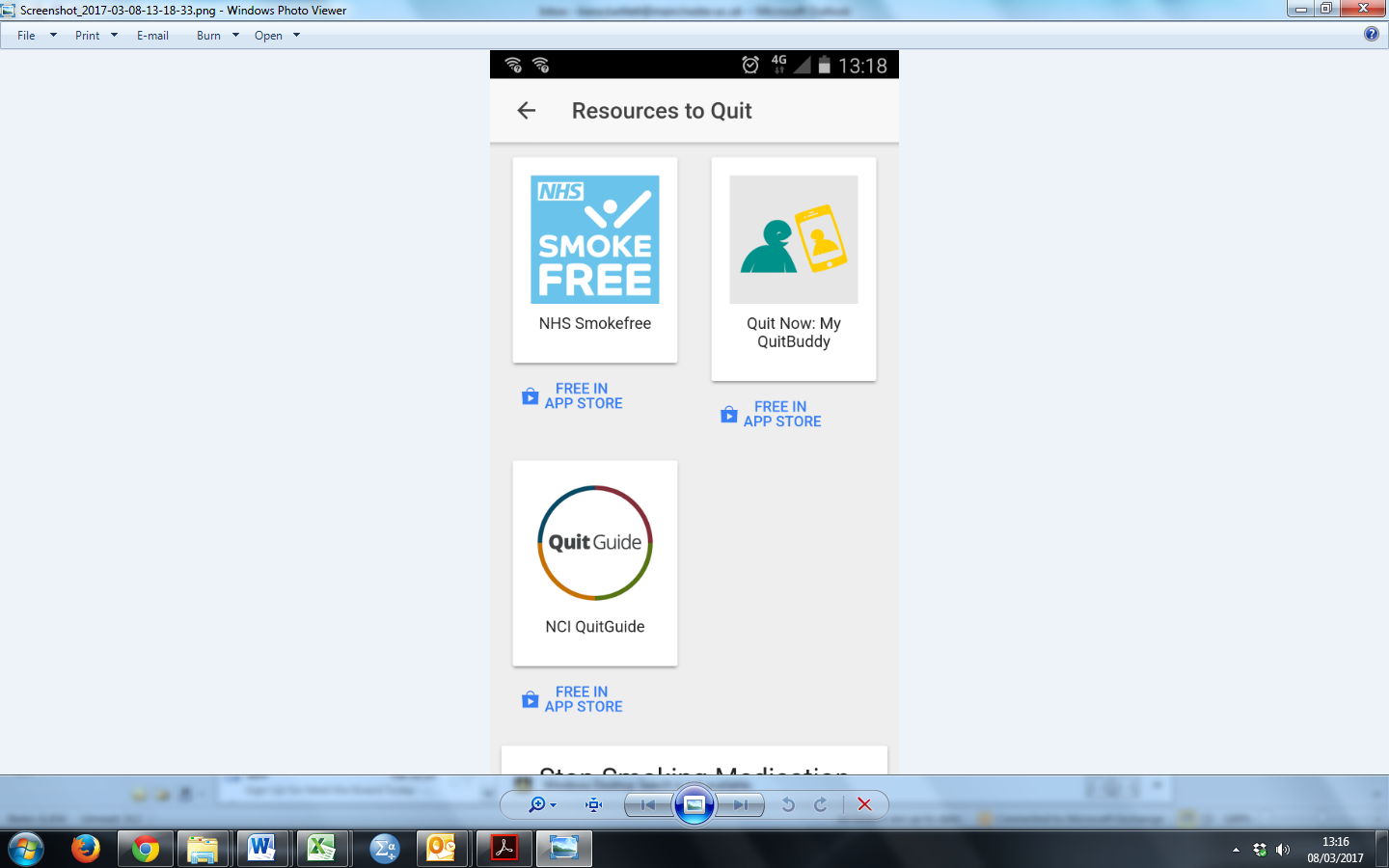 | NHS Smokefree  <http://www.nhs.uk/Tools/Pages/smokefree.aspx> |
| --- | --- |
| 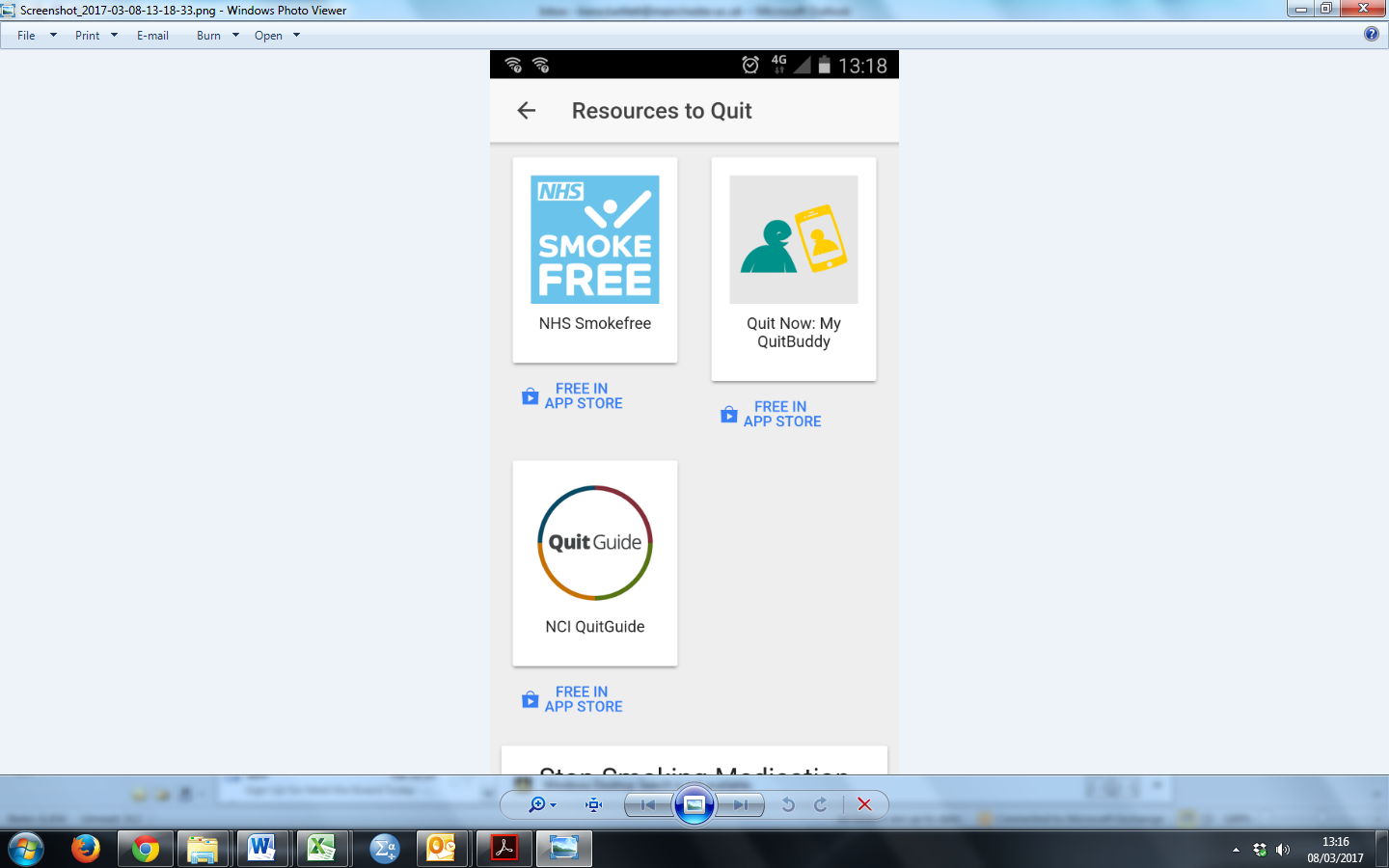 | Quit Now: My QuitBuddy  <http://www.quitnow.gov.au/internet/quitnow/publishing.nsf/Content/quit-buddy> |
| 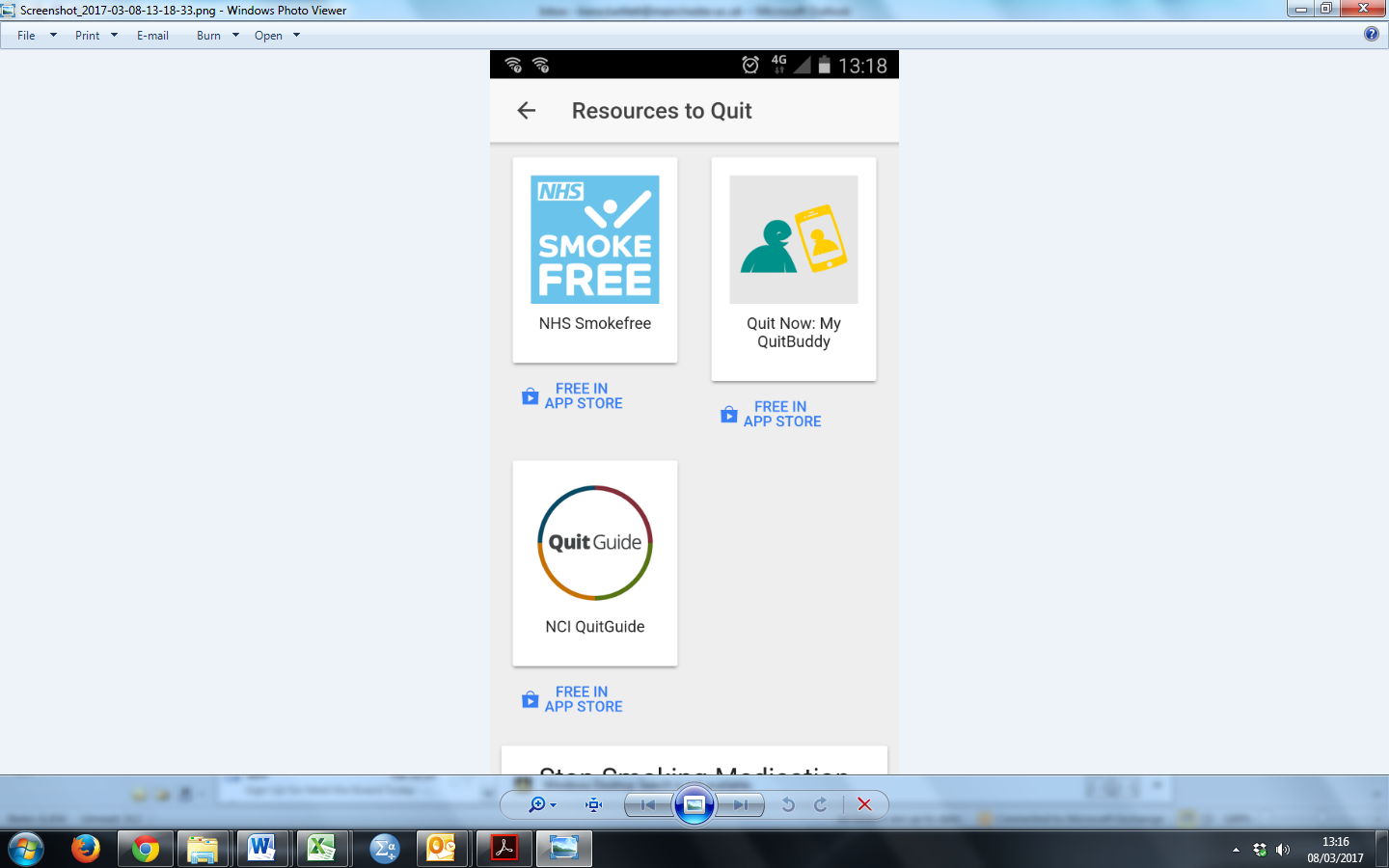 | NCI QuitGuide  <https://www.cdc.gov/tobacco/campaign/tips/quit-smoking/mobile-quit-guide/index.html> |

Stop Smoking medication

Nicotine replacement therapy, or tablet medications? Find out more about the options available below.

Nicotine replacement therapy doubles your chance of quitting. It’s much safer than smoking, and cheaper too! All the below options are available in a range of strengths

- Nicotine gum is available in a range of flavours, you need to chew the gum at first, then park it between your cheek and gum to get quick release nicotine – this is good if you’re experiencing a craving
- Nicotine patches provide nicotine through the skin, they release nicotine slowly. Some are worn all the time and some should be taken off at night
- Nicotine inhalators look like plastic cigarettes with a nicotine cartridge. They are only available on prescription so you would need to speak to your GP or stop smoking advisor if you wanted to give one of these a try
- Nicotine lozenges and strips release nicotine as they dissolve in your mouth. The strip dissolves quickly and the lozenge takes 20- 30 minutes

Speak to your Dr about using more than one nicotine replacement therapy at once

Tablet medications

Contact your GP or stop smoking advisor about the medications available to help you quit smoking

- Champix works in two ways 1) it reduces cravings for nicotine and 2) it blocks the rewarding and reinforcing effects of smoking.
- Zyban was originally developed to treat depression, but has since been found to help people quit smoking. It is thought Zyban has an effect on the parts of the brain that are involved in addiction

E-Cigarettes

There is not as much research supporting the use of e-cigarettes to quit smoking as there is for other treatments such as NRT, tablet based medications and behavioural support from trained advisors. If you are interested in finding out more, visit:

<http://www.nhs.uk/Livewell/smoking/Pages/e-cigarettes.aspx>

Weight Gain

To find out more about weight gain after quitting smoking, and how to manage it visit: <http://www.nhs.uk/Livewell/smoking/Pages/weightgain.aspx>

Myth-busting

The NHS has a page dedicated to busting myths around smoking and quitting <http://www.nhs.uk/Livewell/smoking/Pages/stop-smoking-treatment-myths.aspx>

Health Talk Online

Health talk online is a resource where people tell their own stories through video

http://www.healthtalk.org/peoples-experiences/healthy-living/giving-smoking/overview

**Smokers’ Health Study**

**Online consent form**

Please read the following statements, if you have any questions please contact the research team on 0161 3065436

1. I confirm that I have read the information sheet dated 11^th^ April 2017 on the above project and have had the opportunity to consider the information and ask questions and had these answered satisfactorily
2. I understand that my participation in the study is voluntary and that I am free to withdraw at any time without giving a reason
3. I understand I will be asked to complete an online questionnaire before and after the study period
4. I understand that I will be randomised and asked to **either** use the app and answer brief questions **or** complete brief weekly online questionnaires during the study period
5. I understand that if I am asked to use it, my use of the app will be recorded
6. I understand that I may be contacted if I have not used the app for a few days, or not completed an online questionnaire a few days after it has been sent to check for any technical problems
7. I agree that any data collected may be passed to other researchers and others associated with the project, and stored for use in future research studies. My name, address and email address will never be shared with others
8. I understand that I may be withdrawn from the study by the research team
9. I take responsibility for any activities I choose to complete while using the app
10. I agree that I will not use the app, or the online questionnaires, check or respond to notifications if it is unsafe (e.g. while driving)

**By clicking ‘Agree’ you are accepting each of the above statements and are agreeing to take part in the research**

[Agree button], [Decline button]

***
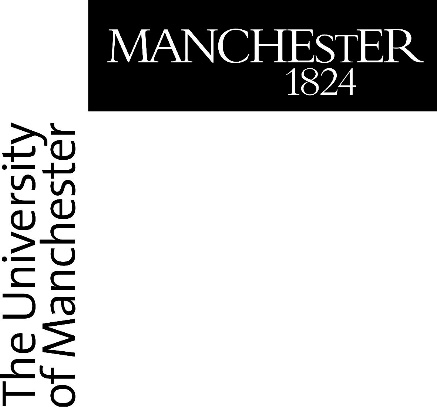
Smokers’ Health Study***

**Participant Information Sheet**

You are being invited to take part in a research study. Before you decide it is important for you to understand why the research is being done and what it will involve. Please take time to read the following information carefully and discuss it with others if you wish. Please ask if there is anything that is not clear or if you would like more information. Take time to decide whether or not you wish to take part. Thank you for reading this.

**What is the aim of the research?**

Researchers at the University of Manchester are recruiting smokers who do not plan to quit smoking, who use smartphones in order to develop an app that focusses on improving well-being of smokers, and provide them with resources to stop smoking should they want to quit at some point. This study will assign participants at random to either using the app or not using the app. All participants will fill out questionnaires at the beginning and the end of the study. The aim of the study is to see how people are using the app, and also to see if there are any changes in mood, motivation to quit, and smoking patterns over time, compared with people who are not using the app.

**What would I be asked to do if I took part?**

If you decide to take part you will be asked to complete a consent form and an online questionnaire, then there is an equal chance you will be asked to either 1) use the app for up to two months, this includes completing questions on the app, planning activities and looking at information or 2) complete weekly questionnaires online for up to two months. After the study has concluded, you will be asked to complete another questionnaire. During the study, a member of the research team may call you a few times to check that you are not having any technical problems with the app, or the questionnaires.

**What happens to the data collected?**

Any personal information such as name, address and telephone number will only be accessible to the research company you have signed up with, and the research team. Personal information will be transferred securely from the research company to the research team and stored in a password protected spreadsheet on a secure, password-protected University of Manchester computer. The answers given to the online questionnaires will be collected and stored in accordance with the Data Protection Act, the data will only be identified with a participant number and this data will not be accessible by the research company. Once the University of Manchester research team have analysed the data we may share summary results with colleagues. The summaries will be associated with participant numbers, so no one reading them will know who took part. If you are asked to use the app, the app will also collect data about the screens you have looked at, your answers to assessments, and the features that you have used. You will not need to put your name into the app, or the online questionnaires. Your data will be identified by ID number only, the data will be stored for use in related research studies in the future, but you will not be contacted by the University of Manchester after the end of the research and your personal information will not be passed to any third party organisation.

**How is confidentiality maintained?**

You will only be identified by a participant number. Once securely transferred from the research company, identifiable information such as name and address will be stored on a password protected spreadsheet accessible only by the research team, and stored in a separate place from the data. All electronic data will be stored on University of Manchester or Boston University computers.

**What happens if I do not want to take part or if I change my mind?**

It is up to you to decide whether or not to take part. If you do decide to take part you will be asked to complete an online consent form. If you decide to take part you are still free to withdraw at any time without giving a reason. You will only be able to withdraw data already collected if you inform the research team within 2 months of completing the online consent form.

**Will I be paid for participating in the research?**

You will not be paid for taking part in the research, but as a thank you we will send a £20 love2shop voucher to you once you complete the final online questionnaire.

**What is the duration of the research?**

The research will last up to two months. The initial questionnaire and questionnaire at the end will take between 15 and 30 minutes each. For those who are allocated to the app group the total time using the app over the two months is estimated at around 2 hours (approximately 20 minutes to sign up and personalise the app, under a minute per day to answer questions and approximately 5 minutes per week to set activities). For those allocated to the online questionnaire group, each of the 8 weekly questionnaires will take around 2 minutes to complete. Phone calls with the research team to introduce the research, discuss which group you have been allocated to and address any technical problems are not expected to take over 30 minutes over the 2 months.

**Where will the research be conducted?**

You will not be asked to attend a specific location, the app will be available to you on your mobile phone and the online questionnaires will be available either on your mobile phone, tablet, or home computer.

**Will the outcomes of the research be published?**

The outcomes of the research may be published in conference proceedings, journal articles or a book chapter. You will not be identifiable from any of the publications.

**Who has reviewed the research project?**

This research has been granted ethical approval by the Manchester Research Ethics Committee 5 (ref: 2017-0128-2113)

**Contact for further information**

You may ask questions about this study at any time. You may contact Dr. Kiera Bartlett the Research Associate on the project on 0161 3065436, or email [kiera.bartlett@manchester.ac.uk](mailto:kiera.bartlett@manchester.ac.uk)

**What if something goes wrong?**

The researchers may choose to withdraw you from the study if there are indications that either using the app, or taking part in the research may be detrimental for your health.

**What if I want to make a complaint?**

**Minor complaints**

If you have a minor complaint then you need to contact the researcher(s) Dr Kiera Bartlett in the first instance, on **0161 3065436,** or email [**kiera.bartlett@manchester.ac.uk**](mailto:kiera.bartlett@manchester.ac.uk)

**Formal Complaints**

**If you wish to make a formal complaint or if you are not satisfied with the response you have gained from the researchers in the first instance then please contact** the Research Governance and Integrity Manager, Research Office, Christie Building, University of Manchester, Oxford Road, Manchester, M13 9PL, by emailing: [research.complaints@manchester.ac.uk](mailto:research.complaints@manchester.ac.uk)  or by telephoning 0161 275 2674 or 275 2046.
